# Can we rely on “calibrated” central venous pressure to measure pleural pressure at the bedside?

**DOI:** 10.1186/s40635-024-00613-y

**Published:** 2024-03-04

**Authors:** Alessandro Protti, Maurizio Cecconi

**Affiliations:** 1 Department of Biomedical Sciences, Humanitas University, Pieve Emanuele, Milan, Italy; 2 Department of Anesthesia and Intensive Care Units, IRCCS Humanitas Research Hospital, Rozzano, Milan, Italy

Measuring the transpulmonary (alveolar–pleural) pressure may be important. However, estimating pleural pressure requires esophageal manometry, which is only available at selected centers. Previous studies have suggested that ventilation-induced changes in central venous pressure (∆CVP) may reflect those in pleural pressure (∆Ppl) [1], but results have been conflicting.

In this issue of the Journal, Kyogoku et al. describe a method for predicting ∆Ppl from ∆CVP after calibrating the two during an occlusion test [2]. The study involved ten mechanically ventilated pigs with acute lung injury and an intrathoracic catheter to measure ∆Ppl directly (reference technique), an esophageal balloon to measure changes in esophageal pressure (∆Pes), and an intrathoracic central venous catheter to measure ∆CVP. The method involves four steps. First, compress the animal’s chest during an end-expiratory airway occlusion maneuver and record the change in airway pressure (∆Paw) and ∆CVP. The lung volume remains constant with an occluded airway, so ∆Paw should reflect ∆Ppl. Second, calculate the calibration coefficient “k” as the ratio of ∆Paw (≈∆Ppl) to ∆CVP. It represents ∆Ppl for each 1-cmH_2_O change in CVP. Third, resume ventilation and record ∆Paw and ∆CVP during an end-expiratory and end-inspiratory occlusion maneuver. Fourth, multiply this ventilation-induced ∆CVP by k to estimate the corresponding (∆CVP-derived) ∆Ppl. This method was also used in animals with low or high intravascular volume or intrabdominal hypertension.

During the occlusion tests, k was 2.2 ± 1.3, suggesting reduced venous return or partial transmission of ∆Ppl to the right heart. During ventilation, ∆Ppl was 7.6 ± 4.5 cmH_2_O, ∆Pes 7.2 ± 3.6 cmH_2_O, and ∆CVP-derived ∆Ppl 8.0 ± 4.8 cmH_2_O. In the Bland–Altman analysis, the bias between ∆Ppl and ∆CVP-derived ∆Ppl was −0.3 cmH_2_O, and between ∆Ppl and ∆Pes, 0.5 cmH_2_O. The 95% limits of agreement (LOA) ranged from −4.1 to 3.4 cmH_2_O and from −2.8 to 3.9 cmH_2_O respectively. These results were consistent across all experimental conditions and indicate that calibrated CVP was accurate (small bias) but not precise enough to be deemed clinically acceptable (wide 95%-LOA and an average percentage error of around 50%) [3]. Of note, during the occlusion test, the positive end-expiratory pressure (PEEP) was 0 cmH_2_O and the lung volume and transpulmonary pressure were constant. During ventilation, PEEP was 6 cmH_2_O, and lung volume and transpulmonary pressure increased. It is possible that lung inflation also affected the ∆CVP and the precision of the estimates, which (instead) assumed a constant k.

∆CVP-derived ∆Ppl was as good or bad as ∆Pes in estimating ∆Ppl, which is surprising. Esophageal manometry was reliable in other studies where ∆Ppl was measured with flexible flat, wafer-type, air-filled balloons [4, 5]. Here it was measured with an intrathoracic method at risk of compression or distortion. In 7/60 occlusion tests, ∆Ppl unexpectedly differed by more than 20% from ∆Paw.

To sum up, using an occlusion test to calibrate the ∆CVP against ∆Paw is a welcome step in measuring transpulmonary pressure. However, the estimates are still too imprecise. Further studies are needed to understand better the intricate impact of changes in intrathoracic pressure on the cardiopulmonary system.
